# Genetic Deletion of GABA_A_ Receptors Reveals Distinct Requirements of Neurotransmitter Receptors for GABAergic and Glutamatergic Synapse Development

**DOI:** 10.3389/fncel.2019.00217

**Published:** 2019-06-04

**Authors:** Jingjing Duan, Saurabh Pandey, Tianming Li, David Castellano, Xinglong Gu, Jun Li, Qingjun Tian, Wei Lu

**Affiliations:** ^1^Synapse and Neural Circuit Research Unit, National Institute of Neurological Disorders and Stroke, National Institutes of Health, Bethesda, MD, United States; ^2^Department of Anatomy and Neurobiology, Zhongshan School of Medicine, Sun Yat-sen University, Guangzhou, China

**Keywords:** GABAergic synapse, GABAergic synapse development, GABA_A_ receptor, cell autonomous, glutamate receptor, inhibitory synapse, excitatory synapse

## Abstract

In the adult brain GABA_A_ receptors (GABA_A_Rs) mediate the majority of synaptic inhibition that provides inhibitory balance to excitatory drive and controls neuronal output. In the immature brain GABA_A_R signaling is critical for neuronal development. However, the cell-autonomous role of GABA_A_Rs in synapse development remains largely unknown. We have employed the CRISPR-CAS9 technology to genetically eliminate GABA_A_Rs in individual hippocampal neurons and examined GABAergic and glutamatergic synapses. We found that development of GABAergic synapses, but not glutamatergic synapses, critically depends on GABA_A_Rs. By combining different genetic approaches, we have also removed GABA_A_Rs and two ionotropic glutamate receptors, AMPA receptors (AMPARs) and NMDA receptors (NMDARs), in single neurons and discovered a striking dichotomy. Indeed, while development of glutamatergic synapses and spines does not require signaling mediated by these receptors, inhibitory synapse formation is crucially dependent on them. Our data reveal a critical cell-autonomous role of GABA_A_Rs in inhibitory synaptogenesis and demonstrate distinct molecular mechanisms for development of inhibitory and excitatory synapses.

## Introduction

GABA_A_ receptors (GABA_A_Rs) are ligand-gated hetero-pentameric anion channels assembled from various combinations of 19 subunits: α (1-6), β (1-3), γ (1-3), δ, 𝜖, 𝜃, π, and ρ (1-3), although most GABA_A_Rs in the brain consist of two α subunits, two β subunits, and one γ or δ subunit (Macdonald and Olsen, 1994; Chang et al., 1996; Sieghart and Sperk, 2002; Mody and Pearce, 2004; Olsen and Sieghart, 2008). These receptors mediate the majority of phasic and tonic inhibition in the adult brain and are critical in the regulation of neuronal excitability and neural network function. In developing neurons, GABA_A_R activation can also provide membrane depolarization and increase neuronal activity, resulting from a relatively positive Cl^-^ reversal potential due to high expression of Na^+^/K^+^/Cl^-^ co-transporter 1 (NKCC1) and low expression of K^+^/Cl^-^ co-transporter 2 (KCC2) in immature neurons (Ben-Ari et al., 1997; Owens and Kriegstein, 2002; Ben-Ari et al., 2007).

The role of GABA_A_R-mediated signaling in neuronal development and function has been extensively studied (Ben-Ari et al., 1997; Owens and Kriegstein, 2002; Ben-Ari et al., 2007). Early pharmacological experiments have demonstrated that GABA_A_R activity can modulate neurogenesis, neuronal migration and differentiation, and synapse development in the immature brain (Owens and Kriegstein, 2002; Ben-Ari et al., 2007). However, pharmacological approaches do not separate the cell-autonomous function of GABA_A_Rs from indirect neuronal network effects associated with global blockade of the receptors, and also do not address the structural role of GABA_A_Rs in the regulation of synapse development. Depolarizing GABA_A_R activity has also been shown to functionally interact with NMDA receptors (NMDARs) to facilitate NMDAR activation and regulate synapse development in immature neurons (Owens and Kriegstein, 2002; Ben-Ari et al., 2007). In addition, by manipulating NKCC1 or KCC2 expression, the role of depolarizing GABA_A_R activity in synapse, and spine development has been inferred (Chudotvorova et al., 2005; Akerman and Cline, 2006; Liu et al., 2006; Wang and Kriegstein, 2008), although recent studies have shown that the regulation of synapse development by KCC2 does not require its ion transport function (Li et al., 2007; Fiumelli et al., 2013). Furthermore, synaptic GABA_A_Rs in knockdown or conventional knockout (KO) mice of GABA_A_R subunits are reduced or lost, leading to the impairment of GABAergic synapse formation and maturation (Fritschy et al., 2006, 2012; Patrizi et al., 2008; Frola et al., 2013), which provides genetic evidence for the role of GABA_A_R subunits in synapse development. However, in these genetic models, neurons may adapt to the global absence of the GABA_A_R subunits and to altered neural network activities throughout their development. Thus, the cell-autonomous role of GABA_A_Rs in the regulation of synapse development remains largely unclear.

Here we have utilized the CRISPR-Cas9 approach to perform single-cell genetic deletion (Incontro et al., 2014) of all functional GABA_A_Rs and examine excitatory and inhibitory synapse development. This is achieved by targeting the β1, β2, and β3 subunits of the GABA_A_Rs in individual hippocampal neurons. These β subunits are required for the receptor assembly and agonist binding (Connolly et al., 1996; Tretter et al., 1997; Baumann et al., 2001; Olsen and Sieghart, 2008; Nguyen and Nicoll, 2018). We found that in neurons lacking GABA_A_Rs, GABAergic synapses are strongly impaired without an accompanying change of excitatory transmission and the spine density. Furthermore, combined genetic deletion of GABA_A_Rs and ionotropic glutamate receptors (iGluRs), including both AMPARs and NMDARs, reveals that signaling mediated by these receptors is critical for inhibitory, but not excitatory, synapse development.

## Materials and Methods

### Mouse Maintenance

All experiments using mice were performed in accordance with animal protocols approved by the Institutional Animal Care and Use Committee at National Institute of Neurological Disorders and Stroke (NINDS) and National Institutes of Health (NIH). Adult C57BL/6 mice were purchased from Charles River, housed and bred under a 12-h circadian cycle. *GRIA1-3^*fl/fl*^GRIN1^*fl/fl*^* mice were generated as described previously (Lu et al., 2013). Animals of either sex were used for the experiments.

### Plasmids

Mouse *GABRB*-1, -2, -3 (Myc-DDK-tagged) was purchased from OriGene (Cat #: MR227185, MR 222938, and MR 222856, respectively). FUGW-Cre-mCherry plasmid was a gift from Roger Nicoll’s lab at UCSF. To screen the β1-3 single-guidance RNA (gRNA) sequences for potential off-target effects, we used the gRNA design target tool^1,2^ . The human codon-optimized Cas9 and chimeric gRNA expression plasmid (pSpCas9 BB-2A-GFP, or PX458) was developed by the Feng Zhang lab at MIT and obtained from Addgene (plasmid #48138). To generate (gRNA) plasmids, a pair of annealed oligos (20 bp) was ligated into the single gRNA scaffold of PX458. The primers used to design the specific gRNA targets were:

*GABRB*1 #5 forward (5′ to 3′) CACCg GCCGCGAGGGCTTCGGGCGT; *GABRB*1 #5 Reverse (5′ to 3′): AAAC ACGCCCGAAGCCCTCGCGGC c;

*GABRB*2 #2 forward (5′ to 3′) CACCg CAGACAGCGGCGATTATTAA; *GABRB*2 #2 reverse (5′ to 3′) AAAC TTAATAATCGCCGCTGTCTG c;

*GABRB*3#2 forward (5′ to 3′): CACCg ACGGTCGACAAGCTGTTGAA; *GABRB*3#2 reverse (5′ to 3′): AAAC TTCAACAGCTTGTCGACCGT c.

For β1-3 gRNAs, the *GABRB*1#5- *GABRB*2#2- *GABRB*3#2 triple gRNA expression unit (U6 promoter + gRNA + scaffold + PolyT tail) was *de novo* synthesized by Genscript and cloned into pSpCas9 BB-2A-GFP (β1-3gRNAs) via AflIII/XbaI sites. To rescue the β1-3 subunit deletion, gRNA resistant β1, 2 or 3 (β1^∗^, β2^∗^, and β3^∗^) plasmids were constructed. Briefly, point mutations in β1-3 gRNA-targeting sites were generated by overlapping PCR and cloned into the pCAGGS-IRES-GFP/mCherry expression plasmid. gRNA resistant β1, 2 or 3 in pCAGGS-IRES-mCherry was co-transfected with β1-3gRNAs. Neurons with both GFP and mCherry fluorescence were used for recording and imaging. All constructs were verified by DNA sequencing.

### Cell Culture and Transfection

HEK293T cells were grown in DMEM (GIBCO) supplemented with 10% fetal bovine serum (FBS) (GIBCO), 1% Pen/Strep, 1% Glutamine, and 1% sodium pyruvate, in a humidified atmosphere in a 37°C incubator with 5% CO_2_. Transfection was performed in 24-well plates with indicated cDNAs using calcium phosphate transfection.

### Dissociated Hippocampal Neuronal Culture

Hippocampal cultures were prepared from E18 time-pregnant mice as previously described (Gu et al., 2016a). Briefly, the mouse hippocampi were dissected out in ice-cold Hank’s balanced salt solution, and digested with papain (Worthington, LK003176) solution at 37°C for 45 min. After centrifugation for 5 min at 800 rpm, the pellet was resuspended in DNase I-containing Hank’s solution, then was mechanically dissociated into single cells by gentle trituration using a pipette. Cells were placed on top of Hank’s solution mixed with trypsin inhibitor (10 mg/ml, Sigma T9253) and BSA (10 mg/ml, Sigma A9647), and centrifuged at 800 rpm for 10 min. The pellet was resuspended in Neurobasal plating media containing 2% B27 supplements and L-glutamine (2 mM). Neurons were plated at a density of 150,000–200,000 cells/well on poly-D-lysine (Sigma P6407)-coated 12 mm glass coverslips residing in 24-well plates for electrophysiology recording, and a lower plating density (100,000–150,000 cells/well) was adopted when neurons were used for immunocytochemistry. Culture media were changed by half volume with Neurobasal maintenance media containing 2% B27 supplements and L-glutamine (2 mM) twice a week.

### Neuronal Transfection

Hippocampal neurons were transfected at day 2–3 *in vitro* (DIV2-3) using a modified calcium phosphate transfection as described before (Li et al., 2017). Briefly, 5 μg total cDNA was used to generate 200 μL total precipitates, which was added to each well at a 40 μL volume (5 coverslips/group). After 2 h incubation in a 37°C incubator, the transfected cells were incubated with 37°C pre-warmed, 10% CO_2_ pre-equilibrated Neurobasal medium, and placed in a 37°C, 5% CO_2_ incubator for 20 min to dissolve the calcium-phosphate particles. The coverslips were then transferred back to the original conditioned medium. The cells were cultured to DIV 14–24 before experiments.

### Immunocytochemistry

Cells grown on coverslips were rinsed with PBS twice and fixed in 4% paraformaldehyde (PFA)/4% sucrose/1x PBS solution for 15 min at RT or 1% PFA in 0.1 M Na-acetate buffer for 13 min at RT, permeabilized and blocked with 0.1% Triton X-100/10% normal goat serum in 1x PBS for 1 h (for surface labeling, cells were incubated in 10% normal goat serum in 1x PBS for 1 h without Triton X-100). Cells were labeled with primary antibodies as follows: anti-β1 (1:500, MABN498, Millipore), anti-β2 (1:800, AB5561, Millipore), anti-β3 (1:1000, 75149, Neuromab), anti-Myc (1:1000, 71D10, cell signaling), anti-gephyrin (1:500, 147021, Synaptic Systems), anti-vGAT (1:1500, 131004, 131002, Synaptic Systems), anti-Neuroligin2 (1:1,000, 129511, Synaptic Systems), GluA1(1:1000, MAB2263, Millipore), anti-PSD-95 (1:1000, 75-028, Neuromab), anti-vGluT1 (1:1000, 135302, Synaptic Systems), anti-GFP (1:10000, Aves labs) in 3%NGS/1x PBS solutions, incubated overnight at 4°C. Cells were washed three times with 1x PBS and then incubated with Alexa Fluor 405, 555 or 647-conjugated IgG for 1 h. Coverslips were washed for four times with 1× PBS and mounted with Fluoromount-G (Southern Biotech).

### Image Acquisition and Analysis

Fluorescence images were obtained with the Zeiss Zen acquisition software and a Zeiss LSM 880 laser scanning confocal microscope using a 63 × oil objective (numerical aperture 1.4) at room temperature. Optical sections, merged by maximum projection, were analyzed at a time using the Image J puncta analyzer program. Thresholds were set at 3 SDs above the mean staining intensity of six nearby regions in the same visual field. Thresholded images present a fixed intensity for all pixels above threshold after having removed all of those below. Labeled puncta were defined as areas containing at least four contiguous pixels after thresholding. For puncta analysis, Images from 3 dendrites (35 μm in length) per neuron were collected and quantified. For co-localization analysis, images from soma, or three secondary dendrites (35 μm in length per dendrite) per neuron were collected and quantified. For β subunit co-localization with synaptic markers, β subunits and gephyrin or vGAT were separately thresholded and confirmed visually to select appropriate clusters following a minimal size cut-off, which included all recognizable clusters. The gephyrin- or vGAT- positive β subunit puncta indicate the number of β subunit puncta per 10 mm showing at least 50% pixel overlapping with thresholded gephyrin or vGAT puncta. Synaptic vs. Total ratios was calculated by the measurement of gephyrin- or vGAT- positive β subunit puncta compared to total β subunit puncta. For gephyrin and vGAT co-localization in dendrites, gephyrin and vGAT puncta were separately thresholded and confirmed visually to select appropriate clusters following a minimal size cut-off, which included all recognizable clusters. The gephyrin-positive vGAT puncta indicate the number of vGAT puncta showing at least 50% pixel overlapping with thresholded gephyrin puncta and the co-localization percentage was calculated by the measurement of gephyrin-positive vGAT puncta compared to total number of thresholded vGAT puncta. The same procedure was used to calculate vGAT-positive gephyrin. For spine density analysis, GFP was immunolabeled with anti-GFP antibodies to boost the fluorescence. 2–3 secondary or tertiary dendrites (50–200 μm long, 20–100 μm from the soma) from each neuron were collected, the number of dendritic protrusions were counted manually. For spine type analysis, images from three dendrites (35 μm in length per dendrite) per neuron were collected and spine types in each dendrite were quantified. Different spine types (mushroom, thin, stubby, and filopodia) were counted manually for each dendrite, and the data were combined from three dendrites to calculate the fractions of each type of spines for that neuron.

### Electrophysiology

Electrophysiological recordings were performed in dissociated hippocampal neuronal cultures as described (Gu et al., 2016b). Briefly, recordings were performed in artificial cerebrospinal fluid (ACSF) containing (in mM) NaCl 119, KCl 2.5, NaHCO_3_ 26, Na_2_PO4 1, glucose 11, CaCl_2_ 2.5, MgCl_2_ 1.3. The intracellular solution for mIPSC recording contained (in mM) CsMeSO_4_ 70, CsCl 70, NaCl 8, EGTA 0.3, HEPES 20, MgATP 4, and Na_2_GTP 0.3. The intracellular solution for mEPSC recording contained (in mM) CsMeSO_4_ 135, NaCl 8, HEPES 10, Na_3_GTP 0.3, MgATP 4, EGTA 0.3, QX-314 5, and spermine 0.1. Osmolality was adjusted to 285–290 Osm and pH was buffered at 7.25–7.35. For recording AMPA mEPSCs at -70 mV, both picrotoxin (0.1 mM) and TTX (0.5 μM) were added to ACSF; for recording NMDA mEPSCs at +40 mV, glycine (1 μM), NBQX (10 μM), picrotoxin (0.1 mM) and TTX (0.5 μM) were added to ACSF; for recording mIPSCs at -70 mV, NBQX (10 μM), AP-5 (50 μM), and TTX (0.5 μM) were added to ACSF. In KCl treated experiment, 15mM KCl was applied to depolarize the cultured neuron. For the recording of GABA_A_ receptor mediated tonic current, bicuculline (20 μM) was applied for about 1–2 min in ACSF containing NBQX, AP-5, and TTX. The change of currents was measured at -60 mV. mIPSCs, or mEPSCs were semiautomatically detected by offline analysis using in-house software in Igor Pro (Wavemetrics) developed in Dr. Roger Nicoll’s laboratory at UCSF. All events were visually inspected to ensure they were mI/EPSCs during analysis and those non-mI/EPSC traces were discarded. Series resistance was monitored and not compensated, and cells in which series resistance varied by 25% during a recording session were discarded. Synaptic responses were collected with a Multiclamp 700B amplifier (Axon Instruments), filtered at 2 kHz and digitized at 10 kHz. All pharmacological reagents were purchased from Abcam, and other chemicals were purchased from Sigma.

### Statistics

All data were presented as mean ± sem (standard error of mean). Direct comparisons between two groups were made using two-tailed Student’s *t*-test. Multiple group comparisons were made using one-way analysis of variance (ANOVA) with the Bonferroni test. The significance of cumulative probability distributions was assessed by Kolmogorov-Smirnov test. Statistical significance was defined as *p* < 0.05, 0.01, 0.001, or 0.0001 (indicated as ^∗, ∗∗, ∗∗∗^, or ^∗∗∗∗^, respectively). *p* values ≥ 0.05 were considered not significant (ns).

## Results

We first examined the expression and subcellular distribution of GABA_A_R β subunits (*GABRB*s) in mouse primary hippocampal neuron cultures. In 18 days *in vitro* (DIV) neurons in culture, the immunolabeling of GABA_A_R β subunits, including β1, β2 and β3, demonstrated that all three β subunits were expressed in hippocampal neurons with substantial synaptic localization at both dendritic (Supplementary Figures S1A,B) and somatic (Supplementary Figures S1C,D) regions, consistent with a recent electron microscopy study in rat hippocampi (Kerti-Szigeti and Nusser, 2016). Our data also indicate that among the three β subunits, β2 exhibits a higher level of synaptic distribution (Supplementary Figure S1).

**FIGURE 1:**
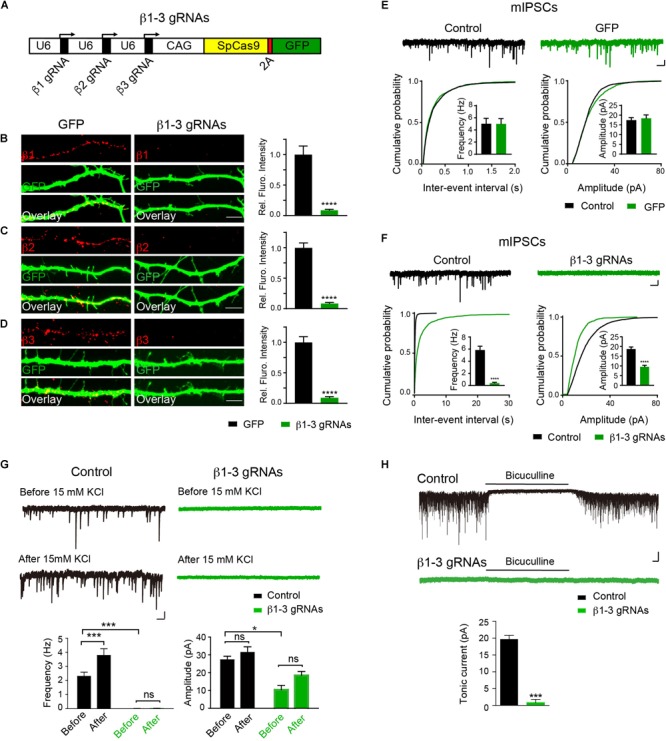
Single-cell KO of the GABA_A_R β1-3 subunits eliminated GABAergic synaptic transmission. **(A)** Schematic of β1-3 gRNA vector. **(B–D)** Representative images showed the loss of β1 **(B)**, β2 **(C)**, or β3 **(D)** subunits in hippocampal neurons expressing β1-3 gRNA vector as compared to neurons expressing the empty gRNA vector (GFP) (GFP, *n* = 15; β1-3 gRNAs, *n* = 15; ^∗∗∗∗^*p* < 0.0001, *t*-test; *N* = 3). Scale bar, 5 μm. **(E)** Expression of the empty gRNA vector (GFP) in hippocampal neurons did not change inhibitory synaptic transmission (control, *n* = 10; GFP, *n* = 9; *N* = 3; *t*-test; *p* > 0.05 for mIPSC frequency and amplitude; Kolmogorov-Smirnov test was used for cumulative graphs, *p* > 0.05 for both conditions). Scale bar, 500 ms, 20 pA. **(F)** Expression of β1-3 gRNA vector in hippocampal neurons essentially eliminated inhibitory synaptic transmission (control, *n* = 19; β1-3 gRNAs, *n* = 21; *N* = 5; *t*-test, ^∗∗∗∗^*p* < 0.0001 for both frequency and amplitude; Kolmogorov-Smirnov test was used for cumulative graphs, ^∗∗∗∗^*p* < 0.0001 for both conditions). Scale bar, 500 ms, 20 pA. **(G)** 15 mM KCl significantly increased the mIPSC frequency in control neurons but not in neurons expressing β1-3 gRNAs at DIV 12-15 (control, ^∗∗∗^*n* = 16; *p* < 0.001 for frequency, *p* > 0.05 for amplitude; β1-3 gRNAs, *p* > 0.05 for frequency, *p* > 0.05 for amplitude; *n* = 19; For β1-3 gRNA amplitude analysis, only 5 out of 19 cells had mIPSC events for analysis; *N* = 2; one-way ANOVA followed by the Bonferroni test). Scale bar, 500 ms, 20 pA. **(H)** GABA_A_R-mediated tonic currents in control and β1-3 gRNAs-expressing neurons at DIV 14-17 (Control, *n* = 23; β1-3 gRNAs, *n* = 15; *N* = 3; ^∗∗∗^*p* < 0.001, *t*-test). Scale bar, 10 s, 50 pA. n represents the number of cells analyzed and N represents the number of independent experiments.

Expression and synaptic localization of β1-3 subunits in hippocampal neurons indicate that functional KO of GABA_A_Rs requires genetic deletion of all three β subunits. To this end, we have employed the CRISPR-Cas9 technology to develop single-guide RNAs (gRNAs) to target gene loci of three *GABRB*s, respectively, that encode GABA_A_R β subunits in mouse genome. Among several gRNA candidates for each β subunits, positive gRNAs that also expressed GFP effectively reduced the expression of co-transfected Myc-β1, 2 or 3 in HEK293T cells (Supplementary Figure S2). We thus generated a construct (hereafter β1-3 gRNAs) containing the three positive gRNAs for β1, β2, and β3, respectively (Figure 1A). To test the KO effect of β1-3 gRNAs in neurons, we transfected hippocampal neuronal cultures at DIV2-3 and performed immunocytochemical analysis at DIV13-14. We found that compared with empty gRNA vector (hereafter GFP)-transfected neurons, the immunolabeling of β1, β2, or β3 in β1-3 gRNAs expressing neurons was strongly diminished (Figure 1B–D). Furthermore, electrophysiological recordings demonstrated that miniature inhibitory postsynaptic currents (mIPSCs) was essentially lost in neurons expressing β1-3 gRNAs (Figure 1F), in agreement with a recent report (Nguyen and Nicoll, 2018). In contrast, no change of mIPSCs was observed in neurons expressing the empty gRNA vector (Figure 1E). Furthermore, we treated the cultured neurons with 15 mM K^+^ to enhance synaptic activity and measured GABAergic transmission. We found that although 15 mM K^+^ significantly increased the mIPSC frequency in control neurons, it didn’t change GABAergic transmission in neurons expressing β1-3 gRNAs (Figure 1G). Indeed, mIPSCs were barely detectable in these neurons either before or after 15 mM K^+^ treatment (Figure 1G). In addition, tonic inhibition generated by persistent activation of extrasynaptic GABA_A_Rs by ambient GABA has been observed in many types of neurons and has a profound effect on neuronal excitability (Farrant and Nusser, 2005; Glykys and Mody, 2007; Belelli et al., 2009). To examine whether extrasynaptic GABA_A_R-mediated tonic currents were diminished in neurons expressing β1-3 gRNAs, we measured tonic currents by adding the GABA_A_R antagonist, bicuculline, in the perfusion solution. We found that compared with control neurons whereby GABA_A_R-mediated tonic currents were readily detected by bicuculline, little tonic currents were observed in neurons expressing β1-3 gRNAs (Figure 1H).

**FIGURE 2:**
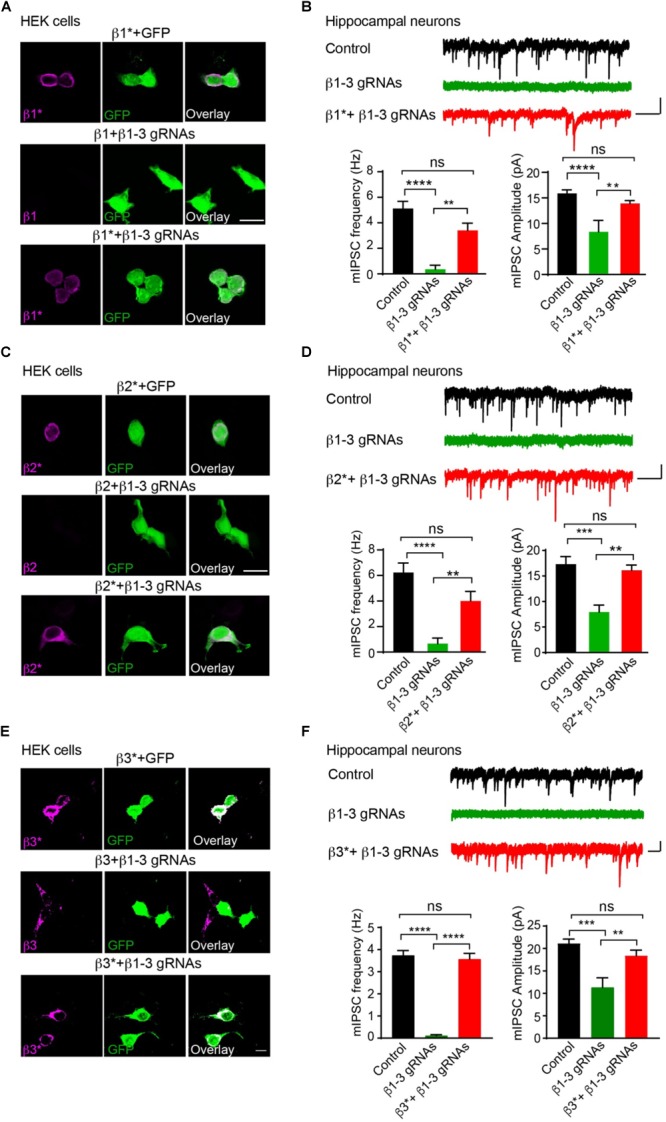
Rescue of GABAergic transmission in neurons expressing β1-3 gRNAs by gRNA resistant β1^∗^, β2^∗^, or β3^∗^. **(A)** Representative images showed that β1-3 gRNA failed to reduce the expression of the gRNA resistant β1 (β1^∗^) in HEK293T cells. Scale bar, 5 μm. **(B)** mIPSC recording showed that β1^∗^ largely rescued the loss of GABAergic transmission in neurons expressing β1-3 gRNAs (*n* = 18, 8, and 15 for control, β1-3 gRNAs, β1^∗^ + β1-3 gRNAs, respectively; *N* = 3; One-way ANOVA followed by the Bonferroni test, ^∗∗^*p* < 0.01, ^∗∗∗∗^*p* < 0.0001). Scale bar, 500 ms, 20 pA. **(C)** Representative images showed that β1-3 gRNA failed to reduce the expression of the gRNA resistant β2 (β2^∗^) in HEK293T cells. Scale bar, 5 μm. **(D)** mIPSC recording showed that β2^∗^ largely rescued the loss of GABAergic transmission in neurons expressing β1-3 gRNAs [*n* = 23, 8, and 12 for control, β1-3 gRNAs, β2^∗^ + β1-3 gRNAs, respectively; *N* = 3; One-way ANOVA followed by the Bonferroni test, ^∗∗^*p* < 0.01, ^∗∗∗∗^*p* < 0.0001 (for frequency), ^∗∗∗^*p* < 0.001 (for amplitude)]. Scale bar, 500 ms, 20 pA. **(E)** Representative images showed that β1-3 gRNA failed to reduce the expression of the gRNA resistant β3 (β3^∗^) in HEK293T cells. Scale bar, 5 μm. **(F)** mIPSC recording showed that β3^∗^ rescued the loss of GABAergic transmission in neurons expressing β1-3 gRNAs (*n* = 12, 12, and 12 for control, β1-3 gRNAs, β3^∗^ + β1-3 gRNAs, respectively; *N* = 3; One-way ANOVA followed by the Bonferroni test, ^∗∗^*p* < 0.01, ^∗∗∗^*p* < 0.001, ^∗∗∗∗^*p* < 0.0001). Scale bar, 500 ms, 20 pA. n represents the number of cells analyzed and N represents the number of independent experiments.

We further performed rescue experiments to characterize the role of individual β subunits in the regulation of GABAergic transmission. To this end, we developed gRNA-resistant β1, β2 or β3 in HEK cells through immunocytochemical experiments (β1^∗^, β2^∗^, or β3^∗^ in Figure 2A,C,E, respectively). We then co-transfected β1^∗^ (Figure 2B), β2^∗^ (Figure 2D), or β3^∗^ (Figure 2F) with β1-3 gRNAs in hippocampal neuronal cultures and measured mIPSCs. We found that the loss of GABAergic transmission in neurons expressing β1-3 gRNAs could be rescued by co-expressing β1^∗^, β2^∗^, or β3^∗^ (Figure 2B,D,F, respectively), indicating that individual β subunits can support basal inhibitory transmission and can substitute for each other for GABAergic transmission in hippocampal neurons. Together, these data demonstrate that we have successfully eliminated GABAergic transmission in single hippocampal neurons and individual β subunits can rescue GABAergic transmission in neurons lacking all endogenous β subunits.

**FIGURE 3:**
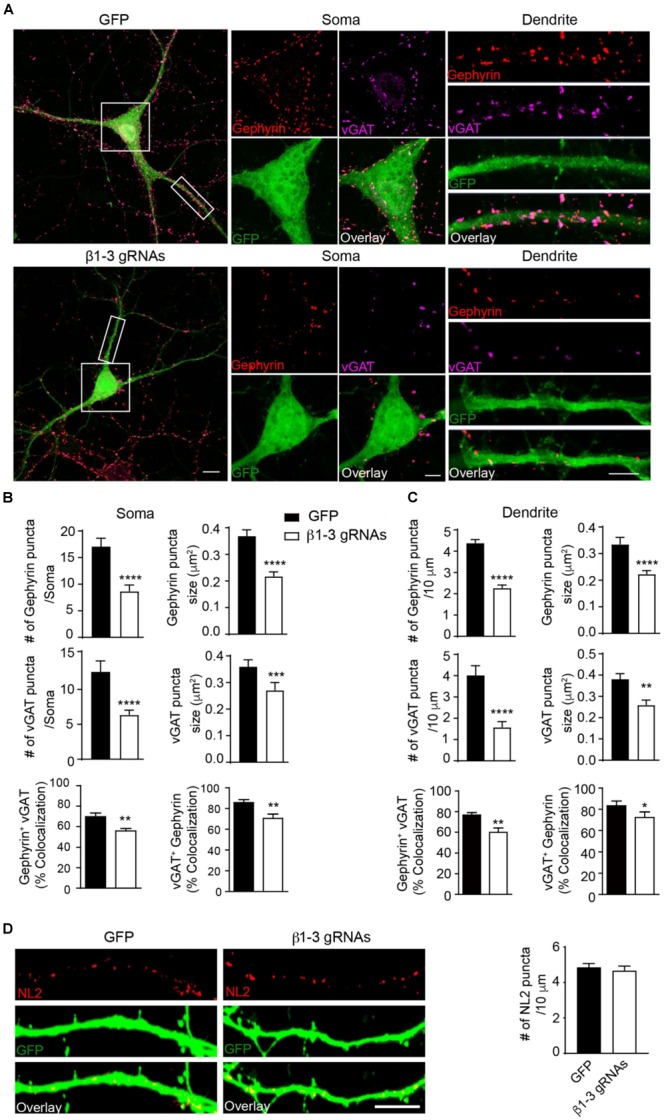
Loss of GABA_A_Rs strongly reduced inhibitory synapse density in hippocampal neurons. **(A–C)** Single-cell KO of GABA_A_Rs significantly reduced gephyrin (red) and vGAT (magenta) puncta as well as co-localization of gephyrin and vGAT in hippocampal neurons. **(A)** Representative images of gephyrin and vGAT-immunolabeling in neurons expressing GFP (top) or β1-3 gRNAs (bottom). **(B)** Bar graphs showed the quantitation of gephyrin (top), vGAT (middle), and co-localization of gephyrin and vGAT (bottom) in neuronal somata (gephyrin density and size: GFP, *n* = 22, β1-3 gRNAs, *n* = 24; ^∗∗∗∗^*p* < 0.0001; vGAT density and size: GFP, *n* = 22, β1-3 gRNAs, *n* = 24, ^∗∗∗∗^*p* < 0.0001, ^∗∗∗^*p* < 0.001; co-localization: percentage of gephyrin puncta colocalized with vGAT (gephyrin^+^ vGAT) (GFP, *n* = 22, β1-3 gRNAs, *n* = 24; ^∗∗^*p* < 0.01) and percentage of vGAT puncta colocalized with gephyrin (vGAT^+^ gephyrin) (GFP, *n* = 22, β1-3 gRNAs, *n* = 24; ^∗∗^*p* < 0.01); *t*-test; *N* = 5). **(C)** Bar graphs showed the quantitation of gephyrin (top), vGAT (middle), and co-localization of gephyrin and vGAT (bottom) in neuronal dendrites (gephyrin density and size: GFP, *n* = 63, β1-3 gRNAs, *n* = 66, ^∗∗∗∗^*p* < 0.0001; vGAT density and size: GFP, *n* = 22, β1-3 gRNAs, *n* = 24, ^∗∗∗∗^*p* < 0.0001, ^∗∗^*p* < 0.01; co-localization: percentage of gephyrin puncta colocalized with vGAT (gephyrin^+^ vGAT) (GFP, *n* = 35, β1-3 gRNAs, *n* = 36; ^∗∗^*p* < 0.01) and percentage of vGAT puncta colocalized with gephyrin (vGAT^+^ gephyrin) (GFP, *n* = 35, β1-3 gRNAs, *n* = 36; ^∗^*p* < 0.05); *t*-test; *N* = 6). GFP was not immunolabeled with anti-GFP antibodies. Scale bar, 10 μm for whole cell, 5 μm for somata and dendrite. **(D)** Neuroligin2 (NL2) puncta were not changed in hippocampal neurons expressing β1-3 gRNAs (GFP, *n* = 19, β1-3 gRNAs, *n* = 21, *p* > 0.05, *t*-test; *N* = 3). GFP was immunolabeled with anti-GFP antibodies to boost the fluorescence (green). Scale bar, 5 μm. n represents the number of cells analyzed and N represents the number of independent experiments.

The loss of functional GABA_A_Rs in individual hippocampal neurons allowed us to examine the cell-autonomous role of these receptors in the regulation of GABAergic synapse development. We found that in neurons expressing β1-3 gRNAs, the immunolabeling of vGAT and gephyrin, the pre- and post-synaptic markers of GABAergic synapses, respectively, at both somatic and dendritic regions, was significantly decreased by ∼50% (Figure 3A–C), indicating a reduction of GABAergic synapse density. Interestingly, there was no change of the puncta density of Neuroligin2 (NL2), a key synaptogenic cell adhesion molecule for GABAergic synapses (Graf et al., 2004; Varoqueaux et al., 2004; Chih et al., 2005; Poulopoulos et al., 2009; Li et al., 2017), in neurons lacking GABA_A_Rs (Figure 3D), suggesting that NL2 clustering is independent of GABA_A_Rs and may be an upstream event of GABA_A_R-mediated signaling for inhibitory synapse development. Taken together, these data demonstrate that genetic deletion of GABA_A_Rs at the single-cell level leads to a substantial reduction of GABAergic synapses.

**FIGURE 4:**
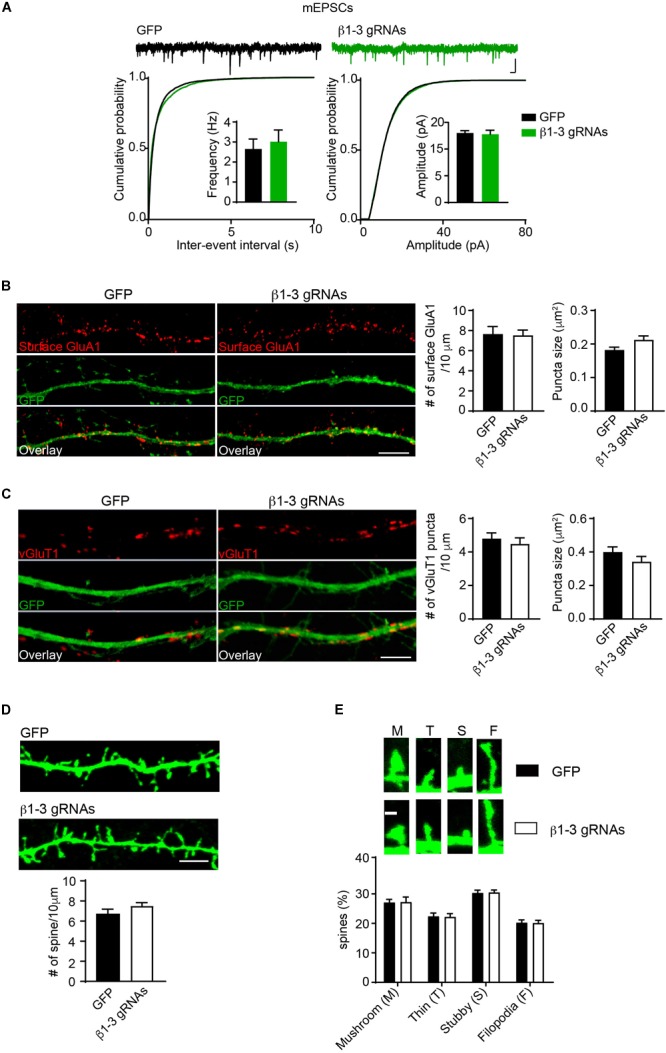
Loss of GABAergic transmission in individual neurons did not change glutamatergic transmission. **(A)** mEPSCs recording showed loss of GABA_A_Rs in individual neurons did not change glutamatergic transmission. Hippocampal neurons were transfected with β1-3 gRNAs at DIV3 and recorded at DIV14-17 (*n* = 25 for GFP and β1-3 gRNAs; *p* > 0.05 for mEPSC frequency and amplitude, *t*-test; Kolmogorov-Smirnov test was used for cumulative graphs, *p* > 0.05 for both conditions, *N* = 4). Scale bar, 100 ms, 20 pA. **(B)** Single-cell genetic deletion of GABA_A_Rs did not change the expression levels of surface GluA1 (GFP, *n* = 17; β1-3 gRNAs, *n* = 19; *p* > 0.05 for both conditions, *t*-test; *N* = 3). GFP was not immunolabeled with anti-GFP antibodies. Scale bar, 5 μm. **(C)** Single-cell genetic deletion of GABA_A_Rs did not change the vGluT1 puncta (GFP, *n* = 24; β1-3 gRNAs, *n* = 28; *p* > 0.05 for both conditions, *t*-test; *N* = 3). GFP was not immunolabeled with anti-GFP antibodies. Scale bar, 5 μm. **(D)** The density of dendritic spines was not altered in hippocampal neuron expressing β1-3 gRNAs at DIV 18 (GFP, *n* = 26; β1-3 gRNAs, *n* = 35; *p* > 0.05, *t*-test; *N* = 3). GFP was immunolabeled with anti-GFP antibodies to boost the fluorescence (green). Scale bar, 5 μm. **(E)** Normal spine types in hippocampal neurons expressing β1-3 gRNAs (GFP, *n* = 26; β1-3 gRNAs, *n* = 28; *p* > 0.05, *t*-test; *N* = 3). GFP was immunolabeled with anti-GFP antibodies to boost the fluorescence (green). Scale bar 1 μm. n represents the number of cells analyzed and N represents the number of independent experiments.

Pharmacological blockade of GABA_A_Rs can induce homeostatic adaptation of excitatory synaptic transmission in neurons (Turrigiano et al., 1998; Shepherd et al., 2006; Anggono et al., 2011; Diering et al., 2014). Specifically, chronic inhibition of GABA_A_Rs reduces AMPAR-mediated excitatory transmission. We thus examined how excitatory synapses adapted to the single-cell silencing of GABAergic inhibitory transmission. Surprisingly, recording of miniature excitatory postsynaptic currents (mEPSCs) in the presence of TTX in hippocampal neurons expressing β1-3 gRNAs showed that there was no change of frequency and amplitude of mEPSCs (Figure 4A). In addition, immunocytochemical analysis demonstrated that the surface expression of GluA1, a key AMPAR subunit in hippocampal neurons (Lu et al., 2009), and the puncta of vesicular glutamate transporter 1 (vGluT1) did not change in β1-3 gRNA-expressing neurons (Figure 4B,C), suggesting that AMPAR trafficking to the neuronal surface and excitatory synapse density were not altered in these neurons lacking functional GABA_A_Rs. Furthermore, we measured the spine density by immunolabeling GFP and found that the spine density or type was not significantly changed in neurons expressing β1-3 gRNAs (Figure 4D,E). Collectively, these data show that single-cell elimination of GABAergic transmission does not induce a homeostatic reduction of excitatory synaptic transmission and also leads to little change of cell biological properties of excitatory synapses, including surface AMPAR expressing levels, and the density of excitatory synapses.

**FIGURE 5:**
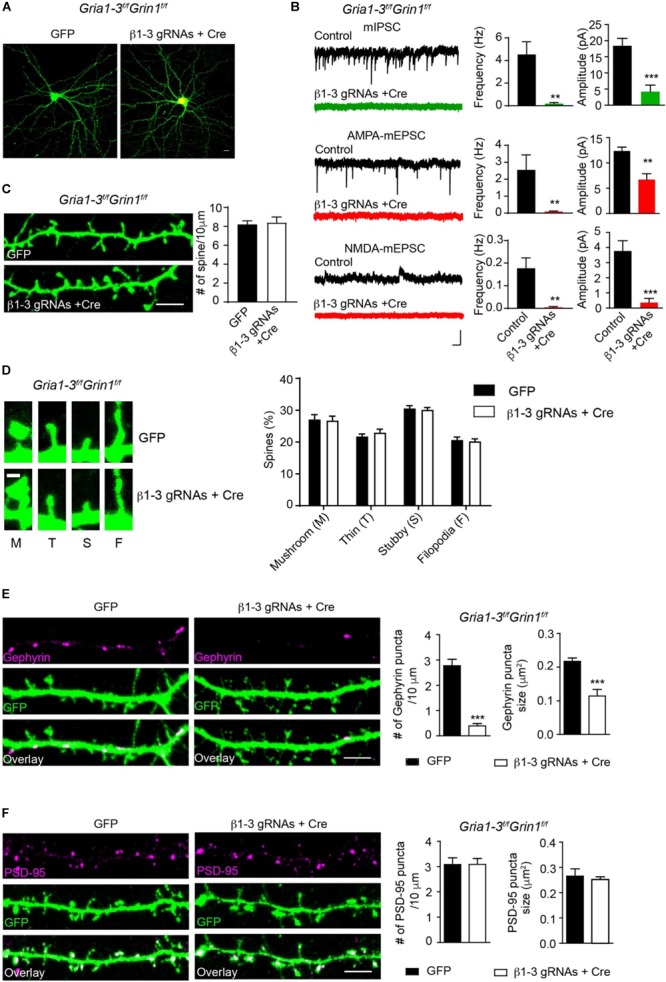
Genetic deletion of GABA_A_Rs, AMPARs, and NMDARs impaired inhibitory, but not excitatory synapses. **(A)** Representative images showed neurons cultured from *GRIA1-3^fl/fl^GRIN1^fl/fl^* mice expressing empty gRNA vector (GFP, left) or expressing both Cre-mCherry/ β1-3 gRNAs(right). Scale bar, 10 μm. **(B)** Representative traces and bar graph showed the loss of GABA_A_R mIPSC, AMPAR-, or NMDAR- mediated mEPSCs in Cre/GFP-positive neurons (mIPSC: control, *n* = 7; Cre + β1-3 gRNAs, *n* = 7; frequency: ^∗∗^*p* < 0.01; amplitude: ^∗∗∗^*p* < 0.001; AMPA mEPSC: control, *n* = 9; Cre + β1-3 gRNAs, *n* = 10; frequency: ^∗∗^*p* < 0.01; amplitude: ^∗∗^*p* < 0.01; NMDA mEPSC: control, *n* = 7; Cre + β1-3 gRNAs, *n* = 7; frequency: ^∗∗^*p* < 0.01; amplitude: ^∗∗∗^*p* < 0.001; *t*-test; *N* = 2). Scale bar, 500 ms, 20 pA. **(C)** Spine density in *GRIA1-3^fl/fl^GRIN1^fl/fl^* hippocampal neurons expressing either GFP or β1-3 gRNAs plus Cre-mCherry (GFP, *n* = 20; β1-3 gRNAs + Cre, *n* = 20; *p* > 0.05, t-test; *N* = 3). GFP was immunolabeled with anti-GFP antibodies to boost the fluorescence (green). Scale bar, 5 μm. **(D)** Normal spine types in *GRIA1-3^fl/fl^GRIN1^fl/fl^* hippocampal neurons expressing β1-3 gRNAs and Cre (GFP, *n* = 25; β1-3 gRNAs + Cre, *n* = 26; *p* > 0.05, *t*-test; *N* = 3). GFP was immunolabeled with anti-GFP antibodies to boost the fluorescence (green). Scale bar 1 μm. **(E)** Single-cell genetic deletion of GABA_A_Rs, AMPARs, and NMDARs dramatically reduced the gephyrin puncta for about 90% (GFP, *n* = 28; β1-3 gRNAs + Cre, *n* = 24; ^∗∗∗^*p* < 0.0001 for puncta density; ^∗∗∗^*p* < 0.0001 for puncta size, *t*-test; *N* = 3). GFP was immunolabeled with anti-GFP antibodies to boost the fluorescence (green). Scale bar, 5 μm. **(F)** Single-cell genetic deletion of GABA_A_Rs, AMPARs, and NMDARs did not change the PSD-95 puncta (GFP, *n* = 22; β1-3 gRNAs + Cre, *n* = 31; *p* > 0.05 for both comparisons, *t*-test; *N* = 3). GFP was immunolabeled with anti-GFP antibodies to boost the fluorescence (green). Scale bar, 5 μm. n represents the number of cells analyzed and N represents the number of independent experiments.

We previously employed a Cre-LoxP system to genetically delete both AMPARs and NMDARs in single hippocampal neurons and found that these iGluRs were dispensable for spinogenesis (Lu et al., 2013). One possibility was that in neurons lacking both AMPARs and NMDARs, remaining GABA_A_Rs could generate depolarizing drive in developing neurons and thus might provide activity necessary for spine development. We thus combined β1-3 gRNAs with the conditional KO of both AMPARs and NMDARs to genetically remove functional GABA_A_Rs, AMPARs and NMDARs in individual neurons and examined excitatory and inhibitory synapses. In hippocampal neuronal cultures prepared from *GRIA1-3^fl/fl^GRIN1^fl/fl^* in which three genes encoding AMPAR subunits (GluA1, GluA2 and GluA3) and the gene encoding the NMDAR obligatory subunit, GluN1, are all conditional alleles (Lu et al., 2013), we co-expressed Cre-mCherry and β1-3 gRNAs through transfection. About 2 weeks after transfection, we performed mIPSCs and mEPSCs recordings in transfected neurons and found the loss of inhibitory synaptic transmission and both AMPAR- and NMDAR-mediated excitatory synaptic transmission in these neurons (Figure 5A,B).

We then measured the spine density in neuronal dendrites and observed no change of spine density or type in Cre-positive, β1-3 gRNA-expressing neurons (Figure 5C,D), as compared to control neurons expressing the empty gRNA vector. We also examined inhibitory and excitatory synapses by the measurement of gephyrin and PSD-95 puncta. We found that compared to neurons expressing the empty gRNA vector, co-expression of both Cre and β1-3 gRNAs in *GRIA1-3^fl/fl^GRIN1^fl/fl^* neurons led to a large reduction of the density of gephyrin puncta (∼90%) (Figure 5E). However, there was no difference of the density of PSD-95 puncta between control neurons and neurons lacking GABA_A_Rs, AMPARs and NMDARs (Figure 5F). Therefore, genetic deletion of both GABA_A_Rs and iGluRs impairs the development of GABAergic synapses but does not change the density of spines or glutamatergic synapses.

## Discussion

To study the role of ionotropic GABA_A_Rs in the regulation of GABAergic synapse development, we have utilized the CRISPR-Cas9 technology to genetically delete all three β subunits of GABA_A_Rs in hippocampal neurons. GABA_A_R β subunits are required for the receptor assembly and GABA binding (Connolly et al., 1996; Tretter et al., 1997; Baumann et al., 2001; Olsen and Sieghart, 2008; Nguyen and Nicoll, 2018), and, consistently, we found that single-cell genetic deletion of three β subunits leads to a loss of GABAergic transmission, in agreement with a recent report (Nguyen and Nicoll, 2018). The lack of inhibitory synaptic transmission at the level of individual neurons allowed us to investigate the cell-autonomous role of GABA_A_Rs in the regulation of GABAergic synapse development.

Our data demonstrate that GABA_A_Rs are critical for GABAergic synapse development at the level of single neurons. Indeed, in hippocampal neurons lacking functional GABA_A_Rs in a mosaic fashion, gephyrin, and vGAT puncta at both somatic and dendritic areas are significantly reduced. Our data are consistent with previous reports in which germline KO of α1 subunit of GABA_A_Rs abolished GABAergic transmission in Purkinje cells, and consequently impaired GABAergic synapse formation (Fritschy et al., 2006; Patrizi et al., 2008). Similarly, knockdown or KO of the GABA_A_R subunit, γ2, which is important for synaptic clustering of GABA_A_Rs (Essrich et al., 1998), impaired GABAergic innervation and reduced GABAergic synapse density (Schweizer et al., 2003; Li et al., 2005; Frola et al., 2013). In addition, GABA_A_R activity has been shown to be important for GABAergic synapse formation (Chattopadhyaya et al., 2007; Arama et al., 2015; Oh et al., 2016; Lin et al., 2018). It is worth noting that broad genetic deletion or widespread pharmacological inhibition of GABA_A_R subunits alter neural network activity, and thus does not separate the cell-autonomous function of GABA_A_Rs from the indirect effects on network activity in the regulation of synapse development. Our data thus provide the genetic evidence of a critical cell-autonomous role of GABA_A_Rs for GABAergic synapse development. Currently, the molecular mechanisms underlying the regulation of GABAergic synapse development by GABA_A_Rs remain largely unclear. It has been reported that the GABA_A_Rs interact with the synaptic adhesion molecule, neurexins (Zhang et al., 2010). In addition, GABA_A_Rs may induce Ca^2+^ influx through NMDARs (Owens and Kriegstein, 2002; Ben-Ari et al., 2007) or voltage-gated calcium channels (Oh et al., 2016) to regulate GABAergic synaptogenesis, and may also play a synaptogenic role in GABAergic synapse development (Fuchs et al., 2013; Brown et al., 2016). Interestingly, the puncta density of NL2, a key synaptogenic cell adhesion molecule for GABAergic synapses (Lu et al., 2016), is not altered in neurons lacking GABA_A_Rs, similar to a previous report (Patrizi et al., 2008), suggesting that NL2 may act upstream of GABA_A_R signaling for GABAergic synaptogenesis. Recent studies have identified that NL2 is crucial for synaptic anchorage of GABA_A_Rs through binding to the GABA_A_R-interacting protein, GARLH/LHFPL4 (Davenport et al., 2017; Yamasaki et al., 2017; Wu et al., 2018) and is critical for GABAergic synapse development (Poulopoulos et al., 2009; Li et al., 2017; Panzanelli et al., 2017). Thus, it is plausible that during development NL2 may regulate GABA_A_R clustering, which in turn modulates GABAergic synapse formation and maturation. Interestingly, in GARLH/LHFPL4 KO neurons, both NL2 and GABA_A_R clustering are impaired (Davenport et al., 2017; Yamasaki et al., 2017; Wu et al., 2018), indicating that GARLH/LHFPL4 may function upstream of both NL2 and GABA_A_Rs in the regulation of GABAergic synapse development.

A recent elegant study has also employed the CRISPR-Cas9 technique to eliminate GABAergic transmission in hippocampal neurons, although this work did not examine the role of GABA_A_Rs in inhibitory synapse development (Nguyen and Nicoll, 2018). In this work, both β2 and β3, but not β1, subunits could rescue the loss of GABAergic transmission (Nguyen and Nicoll, 2018). Specifically, β1 subunit rescued ∼45% GABAergic transmission (Nguyen and Nicoll, 2018). In contrast, in our study all three β subunits rescued inhibitory synaptic currents in neurons expressing β1-3 gRNAs with β1 restoring ∼70% mIPSC frequency. Possibilities to explain this discrepancy include the expression levels of recombinant β subunits through different expression techniques (gene-gun mediated transfection vs. calcium phosphate transfection in our study) and different experimental preparations (hippocampal organotypic cultures vs. dissociated neuronal cultures in our study).

One surprising observation from our study is that excitatory synaptic transmission is normal in hippocampal neurons lacking GABA_A_Rs. It has been well-established that chronic pharmacological inhibition of GABA_A_Rs induces homeostatic adaptation of excitatory synapses and reduces AMPAR-mediated synaptic transmission (Turrigiano et al., 1998; Shepherd et al., 2006; Anggono et al., 2011; Diering et al., 2014). However, the role of GABA_A_R-mediated signaling at the single-cell level in the regulation of glutamatergic transmission remained unclear. Our data now demonstrate that at the level of individual neurons, the complete loss of functional GABA_A_Rs does not impair AMPAR-mediated excitatory transmission. Indeed, glutamatergic transmission, the expression levels of surface GluA1, a major AMPAR subunit in hippocampus (Lu et al., 2009), and the number of vGluT1 puncta are not altered in neurons lacking GABA_A_Rs. In addition, our work reveals that GABA_A_Rs are not absolutely required for spinogenesis in hippocampal neurons *in vitro*, as the spine density in neurons lacking GABA_A_Rs is indistinguishable from that in control neurons (Figure 4D). Our data are consistent with early work in α1 KO mice in which IPSCs are lost in cerebellar Purkinje cells, but EPSCs and excitatory synapses are largely intact (Fritschy et al., 2006; Patrizi et al., 2008). In addition, in GARLH/LHFPL4 KO neurons, GABAergic transmission is severely reduced without an accompanying change of glutamatergic transmission (Davenport et al., 2017; Yamasaki et al., 2017). Similarly, single-cell ablation of gephyrin in hippocampal neurons strongly reduces GABAergic transmission, but does not change glutamatergic transmission (Gross et al., 2016). However, it is important to point out that our data do not exclude the possibility that GABA_A_R activation can sufficiently induce spine formation (Oh et al., 2016) and can modulate glutamatergic synapse development (Ben-Ari et al., 2007).

Previous studies have also indicated a role of NKCC1 and KCC2 in the regulation of excitatory synapse and spine development (Akerman and Cline, 2006; Liu et al., 2006; Wang and Kriegstein, 2008). During development, NKCC1 and KCC2 play fundamental roles in determining the neuronal intracellular Cl^-^ concentration and polarity of GABA_A_R action (Owens and Kriegstein, 2002; Payne et al., 2003; Ben-Ari et al., 2007), and regulate GABA_A_R subunit expression (Succol et al., 2012). Through manipulation of NKCC1 or KCC2 expression in neurons, the role of the depolarizing GABA in excitatory synapse development has been proposed (Akerman and Cline, 2006; Liu et al., 2006; Wang and Kriegstein, 2008). Interestingly, recent studies have indicated that the regulation of synapse or dendritic development by KCC2 is independent of its ion transport function (Li et al., 2007; Fiumelli et al., 2013), suggesting that the effect of KCC2 on neuronal development may be independent of GABA action. Future work toward a more complete understanding of depolarizing GABA in excitatory synapse development will be important to our understanding of the molecular mechanisms underlying synaptogenesis.

We have previously employed a Cre-LoxP system to genetically remove iGluRs, AMPARs and/or NMDARs, in individual hippocampal neurons, and found that excitatory synaptic input is not necessary for development of neuronal spines (Lu et al., 2009, 2013). One possibility is that in developing neurons lacking iGluRs, remaining GABA_A_Rs that are activated may generate depolarizing drives and provide activities important for neuronal morphological development. Thus, to further investigate the role of these receptors in excitatory or inhibitory synapse development, we have combined the Cre-LoxP system with the CRISPR-Cas9 approach to genetically target both iGluRs and GABA_A_Rs. We found that in individual hippocampal neurons lacking both iGluRs and GABA_A_Rs, there was no significant change of the spine density and PSD-95 immunolabeling. These data corroborate a series of recent reports that neurotransmitter release, and thus the activation of postsynaptic neurotransmitter receptors, are not required for spine and excitatory synapse development (Verhage et al., 2000; Varoqueaux et al., 2002; Lu et al., 2013; Sando et al., 2017; Sigler et al., 2017). In contrast, we found that there was a nearly 90% reduction of gephyrin puncta in these neurons lacking both iGluRs and GABA_A_Rs. Previously, we have shown that genetic deletion of both AMPARs and NMDARs led to a strong reduction of inhibitory transmission in hippocampal CA1 pyramidal neurons (Lu et al., 2013). Recently we have further shown that the NMDAR, but not the AMPAR, acts as an important molecule for controlling GABAergic synaptogenesis during development (Gu et al., 2016b; Gu and Lu, 2018). Together, these data suggest that NMDARs and GABA_A_Rs may play a synergistic role in the regulation of GABAergic synapse development. Currently, it remains unclear how NMDARs and GABA_A_Rs work together to control the development of GABAergic connections. It is conceivable that in developing, immature neurons, GABA_A_R activity may facilitate NMDAR activation, inducing Ca^2+^ influx and stimulating signaling pathways important for GABAergic synapse development (Owens and Kriegstein, 2002; Ben-Ari et al., 2007; Gu et al., 2016b). It is also possible that GABA_A_Rs and NMDARs may activate parallel pathways to regulate the formation of GABAergic connections. In the future, it will be imperative to determine the sequential action and functional interplay of signaling pathways mediated by NMDARs and GABA_A_Rs in the regulation of GABAergic synaptogenesis. It will also be important to determine how these neurotransmitter receptors functionally interact with cell surface molecules important for GABAergic synaptogenesis to regulate formation of inhibitory connections (Kuzirian and Paradis, 2011; Ko et al., 2015; Lu et al., 2016; Krueger-Burg et al., 2017).

In summary, through a single-cell genetic approach *in vitro* we have provided new insights into the cell-autonomous role of GABA_A_Rs in developing neurons and discovered a dichotomy in the regulation of synapse development by this prominent Cl^-^ channel. While inhibitory synapse development is critically regulated by GABA_A_Rs, establishment of glutamatergic transmission and excitatory synapses are largely independent of GABA_A_R-mediated signaling at the level of individual neurons. Furthermore, we have managed to remove all AMPARs, NMDARs and GABA_A_Rs in single neurons in culture and demonstrated that iGluR- and GABA_A_R-mediated signaling are not essential for spinogenesis. Our data thus suggest that other developmental pathways including neurotropic factor-mediated signaling (Reichardt, 2006; Park and Poo, 2013), guidance cues, and their receptors (Klein, 2009; Shen and Cowan, 2010; Koropouli and Kolodkin, 2014), *trans*-synaptic cell adhesion interactions (Scheiffele, 2003; Dalva et al., 2007; Missler et al., 2012; de Wit and Ghosh, 2016), and other receptors or channels-mediated signaling (Komatsuzaki et al., 2005; Eroglu et al., 2009; Uemura et al., 2010; Lozada et al., 2012a,b; Kozorovitskiy et al., 2015; Sellers et al., 2015) may play key roles in spinogenesis and excitatory synaptogenesis. It is also worth noting that our data were collected in cultured neurons and there are limitations in using *in vitro* models to study synapse development. Thus, future experiments *in vivo* will help further establish the role of GABA_A_Rs in synaptogenesis. Nevertheless, our data demonstrate a remarkable specificity of these ionotropic receptors in mediating signaling important for synapse development *in vitro*. Given the prominent roles of malfunctions of these receptors in the pathogenesis of many neurodevelopmental disorders (Cellot and Cherubini, 2014; Yuan et al., 2015), our data also highlight the importance of understanding the molecular mechanisms for the regulation of GABAergic synapse development by these receptors.

## Ethics Statement

All experiments using mice were performed in accordance with animal protocols approved by the Institutional Animal Care and Use Committee at National Institute of Neurological Disorders and Stroke (NINDS), National Institutes of Health (NIH).

## Author Contributions

JD, SP, and WL designed the experiments. JD, DC, JL, and XG cloned and characterized the gRNA constructs. JD, SP, and TL performed the immunocytochemical experiments. JD performed the electrophysiological assays. QT provided critical technical design and problem solving. JD and WL wrote the manuscript. All authors read and commented on the manuscript.

## Conflict of Interest Statement

The authors declare that the research was conducted in the absence of any commercial or financial relationships that could be construed as a potential conflict of interest.

## Footnotes

1 http://crispr.mit.edu

2 http://www.e-crisp.org
