# Supplementary material for: Genetic Deletion of GABAA Receptors Reveals Distinct Requirements of Neurotransmitter Receptors for GABAergic and Glutamatergic Synapse Development
Source: Front Cell Neurosci. 2019 Jun 4;13:217. doi: 10.3389/fncel.2019.00217 (PMC6558517; doi:10.3389/fncel.2019.00217)
Supplement: Supplementary file 1 [file Data_Sheet_1.pdf]

Genetic deletion of $GABA_A$ receptors reveals distinct requirements of neurotransmitter receptors for GABAergic and glutamatergic synapse development

Jingjing Duan, Saurabh Pandey, Tianming Li, David Castellano, Xinglong Gu, Jun Li, Qingjun Tian, Wei Lu

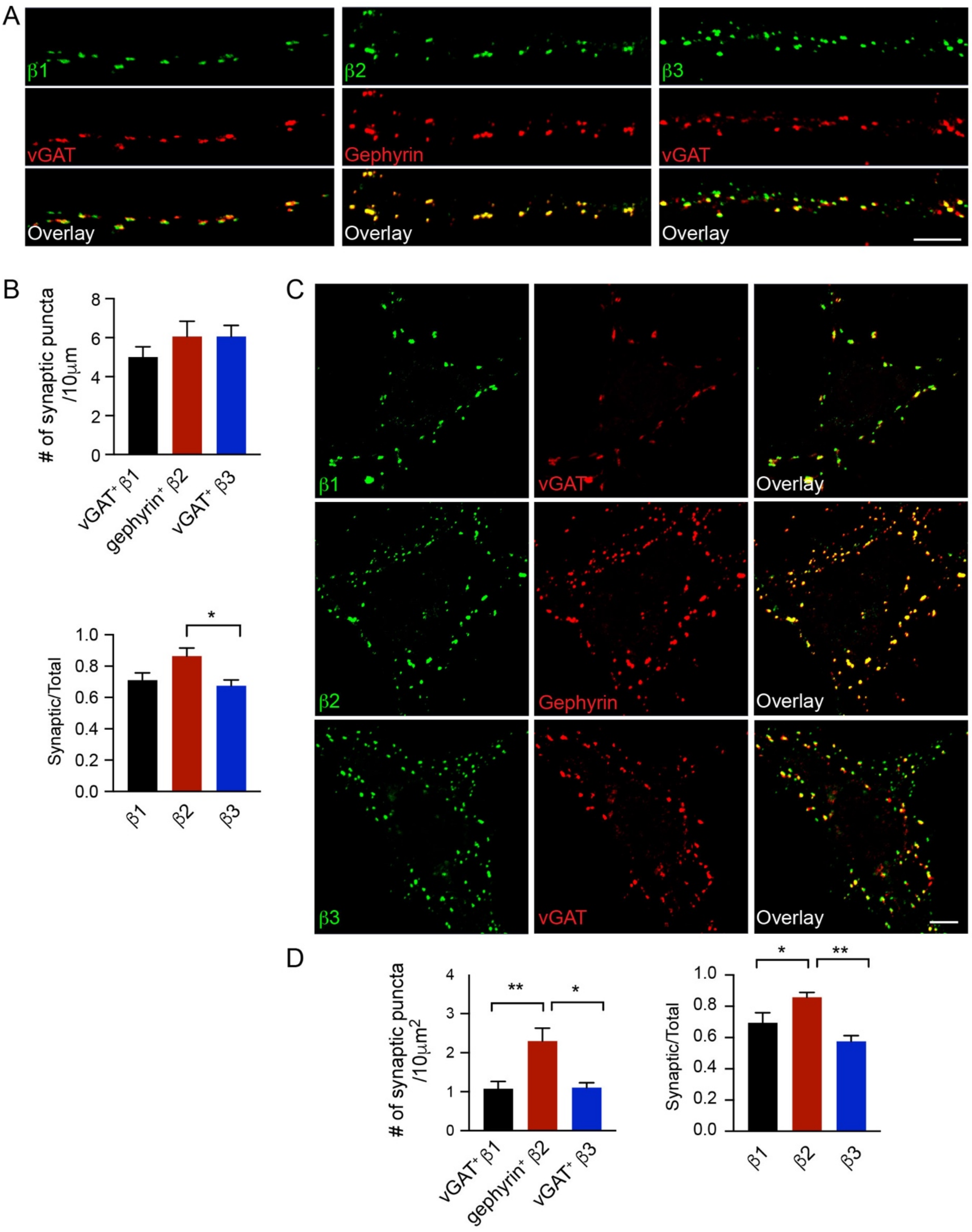

Figure S1. Subcellular distribution of the GABA$_A$R β1-3 subunits in hippocampal neurons.

(A-B) Representative images and summary graphs showed dendrites of neurons at DIV 18 immunolabelled with $GABA_A$ receptor β1, 2, or 3 subunits (green) and synaptic marker gephyrin/vGAT (green). Synaptic $GABA_A$ receptor β subunits (indicated by co-localization with gephyrin or vGAT) was calculated (n = 15; N = 3, *p < 0.05, one-way ANOVA followed by Bonferroni's post-hoc test). Choice of gephyrin or vGAT depends on antibody compatibility. Scale bar, 5 μm.

(C-D) Representative images and summary graphs showed somata of neurons at DIV 18 immunolabelled with $GABA_A$ receptor β1, 2, or 3 subunits (green) and synaptic marker gephyrin/vGAT (green). Synaptic $GABA_A$ receptor β subunits (indicated by co-localization with gephyrin or vGAT) was calculated (n = 10 - 15; N = 3, *p < 0.05, ** p < 0.01, one-way ANOVA followed by Bonferroni's post-hoc test). Choice of gephyrin or vGAT depends on antibody compatibility. Scale bar, 5 μm.

n represents the number of cells analyzed and N represents the number of independent experiments.

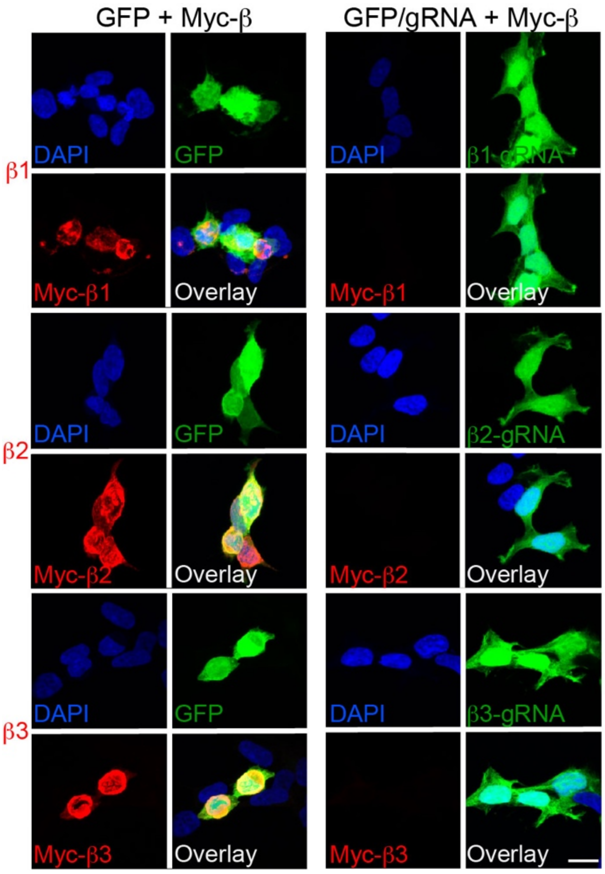

Figure S2. Validation of gRNAs in HEK293T cells

Validation of gRNA-mediated KO of the β1, 2, 3 subunits in HEK293T cells using the CRISPR/Cas9 technique. HEK293T cells were transfected with single β1, 2, 3 gRNAs candidate (also expressing GFP) or GFP alone together with β1, 2 or 3 subunits, respectively. Confocal images showed the loss of β1, 2 or 3 subunits in cells expressing both Myc-tagged β subunits and the respective gRNA candidate (N = 3). Scale bar, 5 µm.

N represents the number of independent experiments.
